# Ovarian cancer recurrence: “is the definition of platinum resistance modified by PARP inhibitors and other intervening treatments?”

**DOI:** 10.20517/cdr.2021.138

**Published:** 2022-06-01

**Authors:** Tanja Pejovic, Katherine Fitch, Gordon Mills

**Affiliations:** ^1^Knight Cancer Institute, Oregon Health & Science University, Portland, OR 97239, USA.; ^2^Department of Obstetrics & Gynecology, Oregon Health & Science University, Portland, OR 97239, USA.

**Keywords:** PARP inhibitors, olaparib, niraparib, ovarian cancer, maintenance therapy

## Abstract

PolyADP ribose polymerase inhibitors (PARPi) have transformed the treatment of ovarian cancer. Particularly in high-grade serous ovarian cancer (HGSOC), a disease characterized by homologous recombination deficiency (HRD), PARPi have had a rapid and profound impact on the disease course, as well as biologic and biomarker definitions of HGSOC, thereby creating a paradigm shift in the approach to treatment. In this review, we discuss the role of PARPi in the maintenance treatment of HGSOC, its effect on platinum sensitivity, and cross-resistance between platinum and PARP inhibitors.

## INTRODUCTION

Ovarian cancer is a chemosensitive disease with chemosensitivity to platinum-based chemotherapy being at least in part due to defects in homologous recombination (see below). However, the majority of the patients recur after platinum-based chemotherapy, typically within 18-24 months of the treatment completion. One of the most reliable predictors of response to subsequent chemotherapy is the duration of progression-free interval (PFI), defined as the interval between completion of the last cycle of platinum-based chemotherapy and the time of disease recurrence (progression)^[1]^. According to Markman’s original observation, the disease that recurs within 6 months of completion of the last platinum-based chemotherapy is considered platinum resistant, whereas recurrence after a PFI of 6 months is considered platinum-sensitive [Table 1]^[1]^. More recently, another category has been introduced - platinum refractory - for the disease that progresses during the platinum-based regimen or within 4 weeks of the last cycle [Table 1]^[2]^. In addition, the partially platinum-sensitive disease was designated as a subgroup of the originally defined platinum-sensitive disease, and it applies to recurrences between 6-12 months from the completion of platinum-based chemotherapy [Table 1]. Initially, the definition of platinum sensitivity applied only to the first recurrence; however, subsequently, the term has been used even beyond 2nd line chemotherapy^[3]^. Platinum sensitivity is based on retrospective clinical observations and some clinicians consider it as a continuum. It is important to know that platinum response remains one of the most critical determinants of clinical management of patients with ovarian cancer, and it is a very important parameter in the design of clinical trials, although there has been some variability in the way the disease categories have been used in trials^[4]^. In general, most patients with ovarian cancer will have a platinum-sensitive disease; this group has a predictable response rate of over 60% to subsequent 2nd line chemotherapy and an expected duration of response of 9-13 months. Patients with partially platinum-sensitive disease, when treated with platinum-based chemotherapy at the time of recurrence, typically achieve a response rate of 39% and median progression-free survival (PFS) of 9.4 months^[5]^. In platinum-resistant disease, 16% (± 12%) of patients can be expected to demonstrate benefit, albeit in most cases with a shorter interval before disease progression^[6]^. Finally, primary platinum-refractory ovarian cancer is uncommon, frequently of non-high grade serous subtype, and has an unfavorable prognosis.

**Table 1:** Platinum sensitivity/resistance classification

**Platinum sensitivity classification**	**Refractory**	**Resistant**	**Partially sensitive**	**Sensitive**
Timing of initial progression	Chemotherapy	0-6 months→	6-12 months→	> 12 months→
Probability of 2nd line platinum response (%)	0	< 10	39	> 60

## PARP INHIBITORS: OVERVIEW

PolyADP ribose polymerase inhibitors (PARPi) have changed the treatment landscape of ovarian cancer in a relatively short time. PARPi initially entered the clinic based on the ability to block base excision repair resulting in the accumulation of double-strand DNA breaks that were synthetically lethal with defects in homologous recombination mediated by mutations in *BRCA1/2*. High-grade serous ovarian cancer has subsequently been demonstrated to have defects in genes involved in homologous recombination DNA repair in at least 50% of cases. Based on these observations, PARPi moved quickly from the laboratory to clinic in the span of 2005 to 2009^[7,8]^. PARPi are approved in ovarian cancer both for treatment of recurrent disease and for maintenance of response to platin agents. Three PARPi have been approved as single-agent therapy for patients who have progressed after multiple prior lines of chemotherapy, showing remarkable activity even late in the disease course. In 2014, olaparib was approved for the 4th line treatment for patients with germline *BRCA *mutations based on the results of Study 42, a single-arm phase 2 study^[9]^. This was followed by the approval of rucaparib as 3rd line treatment for patients with germline and somatic *BRCA *mutations (Ariel 2 and Study 10)^[10,11]^. Finally, in 2019, niraparib was approved in HRD platinum-sensitive late recurrence treatment, with a remarkable response rate of 24% compared with an average 6% response rate in the late recurrent setting (Quadra trial)^[12]^. Subsequent studies have demonstrated activity in earlier stages of therapy and have further demonstrated combination activity with multiple different agents in ovarian cancer. While optimal activity is observed in patients with defects in homologous recombination pathway, there remains a limited activity in patients without HRD as detected by current assays. Whether this represents a failure of current assays to identify all patients with HRD or activity of PARPi outside of HRD remains to be fully elicited.

The success of the PARPi therapy studies in patients with germline and somatic *BRCA *mutated ovarian cancer opened the door to the utilization of PARPi for maintenance in the setting of recurrent platinum-sensitive ovarian cancer. In each of the subsequent 2nd line maintenance studies, PFS was extended for patients with platinum-sensitive disease, with a degree of benefit relative to genetic biomarker status [Table 2]. In SOLO2, a phase 3 study of olaparib maintenance in platinum-sensitive recurrence, there was a PFS difference of 19 *vs*. 5 months in patients with germline *BRCA *mutations receiving olaparib *vs*. placebo^[13]^. In Ariel 3, the PFS doubled from 5.5 to 10.8 months with a hazard ratio (HR) of 0.36 in intention to treat patients with somatic *BRCA* mutation on rucaparib maintenance^[14]^. The Nova trial showed remarkable efficacy of niraparib, but the degree of benefit was relative to biomarker status. Trial participants who received niraparib had a significantly longer median PFS than those in the placebo group in all three pre-specified groups: 21 *vs*. 5.5 months (HR: 0.27) for the germline *BRCA* group; 12.9 *vs*. 3.8 months (HR: 0.38) in the HRD subgroup of the non-germline *BRCA* cohort; 9.3 months *vs*. 3.9 months (HR: 0.45; 95%CI: 0.34 to 0.61) in the overall non-germline *BRCA* mutation cohort^[15]^.

**Table 2:** PARPi maintenance trials

	**Olaparib**	**Niraparib**	**Rucaparib**
**PARPi: first-line maintenance**			
Trial design	• SOLO-1 randomized double-blind Phase 3 study • Trial size: 391 • Olaparib *vs*. placebo	• PRIMA randomized double-blind Phase 3 study • Trial size: 620 • Niraparib *vs*. placebo	
Primary endpoint (mPFS)	BRCAm+ only Not reached at 41 mo *vs*. 13.8 mo	HRD+, 19.16mo *vs*. 8.2 mo (HR: 0.50) BRCAm+ 22.1 mo *vs*. 10.9 mo (HR: 0.40) HRP 8.1 mo *vs*. 5.4 mo (HR: 0.68)	
**PARPi: second-line maintenance**			
Trial design	• SOLO-2 is a randomized double-blind Phase 3 trial • Trial size: 295 • Olaparib *vs*. placebo	• NOVA is a randomized double-blind Phase 3 study • Trial Size: 553 • Niraparib *vs*. placebo	• ARIEL-3 is a randomized double-blind Phase 3 study • Trial size: 564 • Rucaparib *vs*. placebo
Primary endpoint (mPFS)	• Investigator-assessed • (All) 8.4 mo. *vs*. 4.8 mo. (HR: 0.35) Study 19 Data • (BRCAm+) 19.1 mo. *vs*. 5.5 mo. (HR: 0.30)	• Blinded central review • (BRCAwt) 9.3 mo. *vs*. 3.9 mo. (HR: 0.45) • (gBRCAm+) 21.0 mo. *vs*. 5.5 mo. (HR: 0.26)	• Investigator-assessed • (All)10.8 mo. *vs*. 5.4 mo. (HR: 0.36) • (BRCAm+) 16.6 mo. *vs*. 5.4 mo. (HR: 0.23)

HRD: Homologous recombination deficiency; HR: hazard ratio.

After significant success with the use of PARPi in the recurrent setting, and with 80% of patients initially platinum sensitive, PARPi were then explored as first-line maintenance in clinical trials with the hope not only for prolonged PFS, but also the extension of overall survival (OS). Of note, prior clinical trials utilizing chemotherapy with taxol and topotecan as initial maintenance therapy^[16-18] ^showed 8 months PFS advantage, but no impact on OS^[16]^. Bevacizumab maintenance in the up-front setting (GOG 218) has also failed to improve OS^[19]^. Irreversible toxicities of taxanes and bevacizumab include neuropathy, fistula, and stroke. Therefore, prior to moving PARPi to first-line maintenance, most patients with a major response to a platinum analog were in a “watch and wait” period following completion of primary treatment.

SOLO1 changed the landscape of primary maintenance in ovarian cancer^[20]^. In the trial, approximately 400 patients with *BRCA* mutations (germline > somatic) were randomized to receive olaparib or a placebo. After nearly 41 months of follow-up, the treated group had a 70% lower risk of disease progression or death than the placebo group (HR: 0.30). Sensitivity analysis showed absolute longer PFS/PFI with olaparib. The median time to the first subsequent therapy or death was 51.8 months in the olaparib group *vs.* 15.1 months in the placebo group (HR: 0.30; 95%CI: 0.22 to 0.40) [Table 2]. Two other phase 3 trials in frontline maintenance were completed. Olaparib alone was compared to bevacizumab plus olaparib in the Paola-I study, which showed impressive benefit in the intent to treat a population with HR of 0.58^[21]^. In the Prima study within HRD population, the median duration of PFS was 22.1 months in the niraparib group and 10.9 months in the placebo group (HR: 0.40; 95%CI: 0.27 to 0.62) in the subgroup with *BRCA* mutations; in the HRD+ group with no *BRCA* mutation, median PFS was 19.6 months *vs*. 8.2 months in niraparib and placebo groups, respectively (HR: 0.50). In the subgroup of patients with homologous-recombination proficiency, the median duration of PFS was 8.1 months in the niraparib group and 5.4 months in the placebo group (HR: 0.68), leading to FDA approval of niraparib for all patients in the first-line maintenance [Table 2]^[22]^.

## PLATINUM AND PARP INHIBITOR RESISTANCE

As noted above, HRD contributes in part to platinum sensitivity in high-grade serous ovarian cancer. Perhaps the most cogent evidence supporting this contention is the “healing” of defects in *BRCA1/2* in patients treated with platinum analogs. This “healing” reconstitutes HR and contributes to platinum resistance. Given that resistance to PARPi is frequently due to reconstitution of HRD including “healing” of defects in *BRCA1/2*, it is reasonable to assume that PARPi treatment could contribute to resistance to platinum analogs. Furthermore, since patients can receive PARPi maintenance therapy for prolonged periods of time (> 1 year), there is a potential for PARPi to alter the response to retreatment with platinum analogs. In an alternative concept, could the prolonged period of PARPi therapy actually increase the response to platinum retreatment due to the long intervening period? At a minimum, however, the intervening treatment with PARPi requires that we redefine the concept of what period of time from prior platinum treatment would warrant retreatment with a platinum analog rather than moving to a different therapeutic alternative.

In the case of PARPi, the most important issue to address is the question of how sensitive recurrences after maintenance PARPi are to subsequent platinum-based chemotherapy due to the overlap in sensitivity and resistance mechanisms. The initial studies suggest possible cross-resistance between PARPi and platinum^[23]^. MITO, a retrospective study of 234 patients with *BRCA1/2*-mutations, found that patients with progression on olaparib had lower than expected response rates to subsequent platinum therapy, with a response rate of 22% in patients with a PFI > 12 months at the time of recurrence^[24]^. Similarly, Frenel *et al*. reported a secondary analysis of SOLO2 to show that recurrences after olaparib were less sensitive to subsequent platinum treatment compared to patients who received placebo as maintenance, with time to second progression being 14 months *vs*. 7 months in favor of the placebo group^[25]^. Lheureux *et al*. studied 34 patients who had progressed on a prior PARPi and were treated with olaparib and cediranib^[26]^. The study identified mechanisms of resistance among 19 patients: *BRCA*1/2 reversion, *BRCA*1/2 over-expression, multi-drug resistance protein overexpression, and *CCNE*1 amplification/overexpression^[26]^.

Moreover, from ARIEL studies of rucaparib where pretreatment biopsies were required, data showed that patients with BRCA mutation reversions had a shorter PFS with rucaparib than those with no BRCA mutation reversion^[27]^. Other cross-resistance mechanisms to PARPi include (i) *BRCA1 *alternative splicing^[28]^; (ii) *53BP1 *loss^[29]^; (iii) *ABCB1 *gene fusions^[30]^; and (iv) loss of BRCA1 methylation^[27]^.

In patients who progress after olaparib as first-line maintenance, the time to recurrence is crucial to the definition of platinum sensitivity in the context of response to further chemotherapy. This is currently being investigated in the OREO clinical study. Although the initial results suggest that recurrences after a period of at least 24 months may respond favorably to subsequent platinum, additional analyses are needed to precisely discern the degree of platinum sensitivity and particularly the duration of response after PARPi treatment. For example, the reported median PFS in the placebo group of only 2.8 months raises the question of whether a platinum regimen has a very low activity even in responders previously treated with PARPi, or whether the high number of previous lines of therapy in the OREO trial explains the short PFS in a group of patients responding to platinum. Furthermore, one should have a clearer understanding of the degree of benefit from retreatment with a PARPi for patients with *BRCA*-associated tumors whose disease did not progress during PARPi as frontline maintenance compared to patients who were treated with PARPi with subsequent progression. A small study of 22 patients previously treated with PARPi showed that both groups experienced the benefit of retreatment with PARPi, suggesting that the development of resistance is not necessarily universal with prior exposure and progression on PARPi^[31]^.

The complexity of biologic responses to chemotherapy after PARPi maintenance-and to some extent following bevacizumab maintenance as well-has led experts to recommend the use of treatment-free interval (TFI), as opposed to platinum sensitivity status, to more broadly assess whether intervening maintenance agents impact disease response to subsequent treatment^[32]^. It was proposed that TFI be defined as the period from the last disease-directed therapy, including PARPi, platinum-based, and biologic agent treatments (typically bevacizumab)^[32]^. The TFI concept gives us the opportunity to address unanswered questions regarding the length of maintenance treatment with PARPi as first-line maintenance. The current studies have recommended olaparib for 2 years and niraparib for 3 years in frontline maintenance. The time of recurrence and whether the recurrence occurs on treatment *vs*. after completion of prescribed maintenance is associated with the duration of platinum sensitivity. In that sense, it would be important to have a uniform established duration of the first-line maintenance treatment. Finally, there is also a need to determine whether patients who progress on PARPi after an initial response to platinum agents will benefit from retreatment with a platinum analog and to what degree compared to alternative treatment approaches.

As platinum sensitivity may be considered as a continuum, and with maintenance treatment having moved to first-line platinum responders, there is an opportunity to better understand the biological effects of PARPi on the disease response to subsequent therapies. With the response to subsequent therapy being closely related to platinum sensitivity (which is also a marker of PARPi sensitivity), this question merits further investigation via molecular analytics of serial biopsies pre- and post- first line treatment, first-line maintenance and subsequent treatment. The long-term responses in first-line PARPi maintenance treatment may indicate that a group of women will eventually be cured, which would decisively change ovarian cancer treatment and prognosis. However, patients who recur after PARPi or while on PARPi and are retreated with platinum represent the group in which we must obtain additional insight. Given the concept of retreatment with platinum analogs in patients with a prolonged PFI, a number of trials of “PARPi after PARPi” are underway. Even if “PARPi after PARPi” trials yield positive results, combination treatment with PARPi such as PARPi/Wee-i or ATRi/PARPi or PD-1/PD-L1/PARPi approaches have the potential to reverse PARPi resistance and, if toxicity allows them to be moved earlier in the treatment spectrum, may prevent or delay the emergence of PARPi (and potentially platinum) resistance. In this manuscript, we have treated PARPi as a single modality, with this being supported by similar responses in trials across PARPi. However, different PARPi have different trapping abilities and specificity for different members of the PARP family. Further, new PARPi with greater specificity and abilities to cross the blood-brain barrier are being explored. Whether all of the PARPi will have similar effects on platinum sensitivity remains to be determined. We expect that ongoing precise and rigorously designed translational studies will, in the near future, bring more clarity to the best therapy sequence for ovarian cancer patients, and particularly identify populations of patients who are likely to benefit (or not) from platinum analogs following PARPi therapy either therapeutic or maintenance.

## CONCLUSION

Today platinum remains the cornerstone of chemotherapy for ovarian cancer and PARPi are critical as a maintenance treatment. Resistance to platinum and PARPi has important clinical and prognostic significance, and the mechanisms of resistance are being rapidly investigated. A more precise understanding of the genomic markers of HRD, platinum sensitivity, and cross-resistance between PARPi and platinum will require serial biopsies (pre-, on-treatment) to be able to improve patient stratification and identify therapeutic strategies based on molecular vulnerabilities.

## DECLARATIONS

### Copyright

© The Author(s) 2022.
